# The 500^th^ Lung Transplantation at the Faculdade de Medicina da Universidade de São Paulo: Reflecting on Our Journey and Looking Ahead

**DOI:** 10.1590/1516-3180.2023.1414030423

**Published:** 2023-07-07

**Authors:** Flavio Pola dos Reis, Paulo Manuel Pêgo-Fernandes

**Affiliations:** I MD. Attending Physician, Thoracic Surgery Department, Instituto do Coração, Hospital das Clínicas HCFMUSP, Faculdade de Medicina, Universidade de São Paulo, SP, BR.; II MD, PhD. Vice Director, Faculdade de Medicina FMUSP, Universidade de São Paulo, São Paulo, SP, BR; Full Professor, Department of Cardiopulmonology, Instituto do Coração, Hospital das Clínicas HCFMUSP, Faculdade de Medicina, Universidade de São Paulo, São Paulo, SP, BR. Director, Scientific Department, Associação Paulista de Medicina (APM), São Paulo (SP), BR.; Universidade de São Paulo, Faculdade de Medicina, Hospital das Clínicas HCFMUSP, São Paulo, SP, BR; Associação Paulista de Medicina, Scientific Department, São Paulo, SP, BR

Lung transplantation is a widely accepted therapeutic option for the treatment of some advanced lung diseases. With current medical advances and new technologies, discussions on terminal patients and the possibility administering of a treatment option, whose objective is to increase survival or at least the quality of life of patients, are gaining more attention.^1^

## WHERE WE CAME FROM

The modern transplantation era began in the early 1900s, when surgeons Alexis Carrel and Charles C. Guthrie, developed a blood vessel suturing technique and performed experimental transplants. This discovery earned them the Nobel Prize in 1912.^2^

In Brazil, the Faculdade de Medicina da Universidade de São Paulo (FMUSP) was also founded in 1912. The institution is recognized for its excellence in teaching, research, and university extension, and for its pioneering role in solid organ transplantation. In 1965, the institution had its first kidney transplant, which was the second ever in Brazil.^3^ In 1967, at the Instituto do Coração (InCor), Professor Euryclides de Jesus Zerbini performed the first heart transplant in Brazil. However, transplantation techniques only significantly advanced in the 1970s, with the discovery of cyclosporine, the development of a preservation solution, and standardization of organ removal protocols. Due to this progress, the following transplantations programs were created and reactivated at the FMUSP: heart (1984), liver (1985), pancreas (1987), and lung (1989) transplants.^4^

In the 1990s, the discussion on lung transplantation involved adequate donor selection, surgical technique, diagnosis, and treatment of the primary graft dysfunction. In 2003, the progressive increase in the number of transplants led to the creation of an exclusive team to care for and follow-up transplant patients at the FMUSP InCor. Our team includes thoracic surgeons, pulmonologists, infection disease specialists, nurses, physiotherapists, nutritionists, social workers, and psychologists, who help patients on the waiting list and in the postoperative period.

Exactly 110 years after the FMUSP was founded, the Hospital das Clínicas InCor performed its 500^th^ lung transplant, a milestone for thoracic surgery in Brazil and Latin America.

The InCor lung transplant group performed numerous procedures that mark the history and development of transplantation in Brazil. In 2003, we performed the first bilateral lung transplant; in 2006, the first pediatric transplant; in 2011, the first split; and in 2012, the first transplant using the ex-vivo lung perfusion technique. In 2012, we used extracorporeal membrane oxygenation (ECMO) to treat a primary graft dysfunction in a postoperative patient. Currently, 11 years after the transplant, the patient is still being followed-up as an outpatient by the group.^5^

Another important milestone was the creation of Medical Residency in Lung Transplantation for pulmonologists and thoracic surgeons, which was accredited by the National Medical Residency Commission in 2010. Most of our former students currently work in the field of lung transplantation in Brazil.

Currently, Brazil ranks second worldwide among countries that perform the highest number of transplants; moreover, Brazil has the highest public funding for this procedure, as approximately 95% of transplants in the country are funded by the Unified Health System.^3^

Our greatest challenge is to provide health care to patients who have to wait for up to 2 years to get a lung; unfortunately, the natural progression of lung disease does not often allow for such a lengthy waiting time. We began a discussion on how to prioritize patients on the list among transplant groups in the State of São Paulo. However, after analyzing data from the last 10 years, we observed that a key component of the problem was the low organ use rate of less than 5%; in the United States and in Toronto (Canada) the rates are approximately 20% and 30%, respectively.^3^ Another important information is the rate of refusal of relatives to donate organs, which according to the Brazilian Transplant Registry, occurs in up to 42% of cases.

The InCor certainly has the professional and institutional capacity to double the current organ use rate; however, it needs funding and public policies to increase the rate of consent for organ donation, and improve donor management and care.

## LEARNINGS

Telemedicine has been a focal point of discussion in the medical field. Adequate use of technology can favor the population, as we live in a continental country that has few active lung transplantation services. Before the COVID-19 pandemic, lung transplantation groups used telemedicine in two main pillars: the assistant team and the patient. In the assistant team, physicians who needed to discuss cases to assess whether there was an indication for transplantation could easily consult their colleagues. Regarding the patient, contact is first made to assess the case and whether there is an indication for transplantation. If an indication was identified, a face-to-face evaluation was then scheduled.

During the pandemic, there was no discussion about lung transplantation in acute illness. In these cases, the patient’s assistant team had a tele-consultation with a pulmonologist and a thoracic surgeon. If there was an indication, the patient was then transferred to the InCor to commence the specific evaluation.^6^

The management of a patient with terminal lung disease is complex as they have to be evaluated by a multidisciplinary team and, our group believes that a palliative care professional must be present to determine the patient’s therapeutic plan. All patients referred for lung transplant evaluation will also be evaluated by the palliative care group to determine the guidelines. All patients referred for lung transplant evaluation will also be evaluated by the palliative care group to determine the guidelines.^7^

## WHERE ARE WE HEADED

Institutions with a lung transplant program need resources and investments to continue evolving and providing quality care for patients. In 2022, the FMUSP InCor created a biobank, with a capacity of more than 84,000 samples, that could store samples at -80 °C. The progressive use of artificial intelligence and the intersection between the collected materials, associated with retrospective or prospective donor information, has also facilitated personalized care, in addition to being at the frontier of knowledge.

Institutional support is the foundation for the development and continuity of the Transplantation Program. In 2013, an important step was taken with the creation of the InCor Transplantation Center, which included the formation of a multidisciplinary team dedicated to the care of transplant patients. Owing to this structure and support, in 2022, in spite of the COVID-19 pandemic we performed 62 adult heart transplants, 36 lung transplants, and 13 congenital heart transplants, making a total of 111 thoracic organ transplants.

In summary, according to the words of the late Prof. Adib Jatene, “I do not believe in people who save, but in structures that work.” Thus, as a pioneer transplantation institution at national and international levels, this program completed 500 lung transplants with a dedicated, super-specialized, multidisciplinary, and interdisciplinary team that achieved the best possible results, comparable to those of other international groups, and have allowed several patients to return to the society with an improved quality of life.
